# Whole Genome Sequencing and Tn*5*-Insertion Mutagenesis of *Pseudomonas taiwanensis* CMS to Probe Its Antagonistic Activity Against Rice Bacterial Blight Disease

**DOI:** 10.3390/ijms21228639

**Published:** 2020-11-16

**Authors:** Wen-Jen Chen, Tzu-Yen Kuo, Chun-Yi Chen, Feng-Chia Hsieh, Yu-Liang Yang, Je-Ruei Liu, Ming-Che Shih

**Affiliations:** 1 Agricultural Biotechnology Research Center, Academia Sinica, Taipei 11529, Taiwan; willchen@chbio.com.tw (W.-J.C.); tzuyenkuo@gmail.com (T.-Y.K.); chunyic@gate.sinica.edu.tw (C.-Y.C.); ylyang@gate.sinica.edu.tw (Y.-L.Y.); jrliu@ntu.edu.tw (J.-R.L.); 2 Biopesticide Division, Taiwan Agricultural Chemicals and Toxic Substances Research Institute, Council of Agriculture, Taichung 41358, Taiwan; hsiehf@tactri.gov.tw; 3 Institute of Biotechnology, National Taiwan University, Taipei 10617, Taiwan

**Keywords:** *Pseudomonas taiwanensis*, *Xanthomonas oryzae* pv*.oryzae* (*Xoo*), Rice bacterial blight, biocontrol agent

## Abstract

The Gram-negative bacterium *Pseudomonas taiwanensis* is a novel bacterium that uses shrimp shell waste as its sole sources of carbon and nitrogen. It is a versatile bacterium with potential for use in biological control, with activities including toxicity toward insects, fungi, and the rice pathogen *Xanthomonas oryzae* pv*.oryzae* (*Xoo*). In this study, the complete 5.08-Mb genome sequence of *P. taiwanensis* CMS was determined by a combination of NGS/Sanger sequencing and optical mapping. Comparison of optical maps of seven *Pseudomonas* species showed that *P. taiwanensis* is most closely related to *P. putida* KT 2400. We screened a total of 11,646 individual Tn*5*-transponson tagged strains to identify genes that are involved in the production and regulation of the iron-chelator pyoverdine in *P. taiwanensis*, which is a key anti-*Xoo* factor. Our results indicated that the two-component system (TCS) EnvZ/OmpR plays a positive regulatory role in the production of pyoverdine, whereas the sigma factor RpoS functions as a repressor. The knowledge of the molecular basis of the regulation of pyoverdine by *P. taiwanensis* provided herein will be useful for its development for use in biological control, including as an anti-*Xoo* agent.

## 1. Introduction

The genus *Pseudomonas* comprises Gram-negative aerobic gammaproteobacteria which are capable of inhabiting a variety of ecological niches. Its members exhibit great metabolic diversity. Several species benefit plants or degrade environmental pollutants, and some species act as human and plant pathogens, such as *P. aeruginosa* PAO1 and *P. syringae* pv. tomato DC3000 [1]. The genomes of many *Pseudomonas* species have been sequenced, including *P. aeruginosa* PAO1, *P. syringae* pv. *tomato* DC3000, insect pathogen *P. entomophila*, plant promoting growth *P. fluorescens* SBW25 and xenobiotic-compound-degrading bacterium *P. knackmussii* B13 [2,3,4,5,6]. The draft genomes of these *Pseudomonas* species have been used to study potential virulence, regulatory and resistance genes. Genomic background may also provide better understanding of phylogenetic position, horizontal gene transfer, gene organization, and unique genes or islands among *Pseudomonas* species.

Here, we focus on *Pseudomonas taiwanensis* CMS, which is a novel Gram-negative bacterium and displays a board host range. It was isolated from soil in northern Taiwan, and uses shrimp shell waste as the sole carbon and nitrogen source [7,8]. When grown in shrimp shell medium, *P. taiwanensis* could produce high levels of extracellular chitinase, chitosanase and nattokinase [9,10]. It has insecticidal activity against a number of species of Lepidoptera, including vegetable pests *Plutella xylostella*, *Spodoptera exigua,* and *Trichoplusia ni* [8] and a Dipteran species, *Drosophila melanogaster* [9].

In a previous study, we found that *P. taiwanensis* displayed strong antagonistic activity against the rice pathogen *Xoo* [11]. Rice bacterial blight caused by *Xoo* is a major rice disease worldwide, which has been reported in Asia, northern Australia, Africa, the southern part of the United States and Latin America [12]. Rice yield may sustain up to 50% loss in areas with heavy bacterial blight infection [12]. So far, farmers have mostly used chemical pesticides to control bacterial blight disease. However, chemical pesticides cause environmental pollution, soil acidity, and resistance problems. Therefore, development of novel anti-*Xoo* agents will likely be essential in the future.

Previously, we showed that *P. taiwanensis* has anti-*Xoo* activity by the secretion of iron-chelator pyoverdine and that T6SS was found to be involved in the secretion of pyoverdine in *P. taiwanensis* through an unknown mechanism [11]. In this study, we conducted whole genome sequencing of *P. taiwanensis* by high-throughput DNA sequencing (Roche 454 and Illumina Solexa). A combination of 454 and Illumina scaffolds were aligned onto an optical whole genome restriction map (WGRM). The *de novo* assembly contigs from next-generation sequencing were aligned to form large scaffolds and understand their orientation based on an optical WGRM that has been used in many projects [6,13,14]. Transposon can be used to create stable insertion mutant strains which destroy genes function. To dissect the regulatory network of antibacterial action of *P. taiwanensis*, we screened the Tn*5* transposon tag library for *P. taiwanensis* mutants with reduced or increased anti-*Xoo* activity. The flanking sequences of Tn*5* transposon insertion sites were determined and blasted against the genomic sequences of *P. taiwanensis* (NCBI accession no. CP011858), which allowed us to efficiently identified the genes that are mutated by Tn*5* transposon. Taken together, combining data mining of the *P. taiwanensis* draft genome and mutant toxicity data, we were able to precisely identify virulence factors and pathogenic pathways involved in the antagonistic activity against rice pathogen *Xoo*.

## 2. Results and Discussion

### 2.1. Sequencing and Assembly of P. taiwanensis Complete Genome

**Figure 1:**
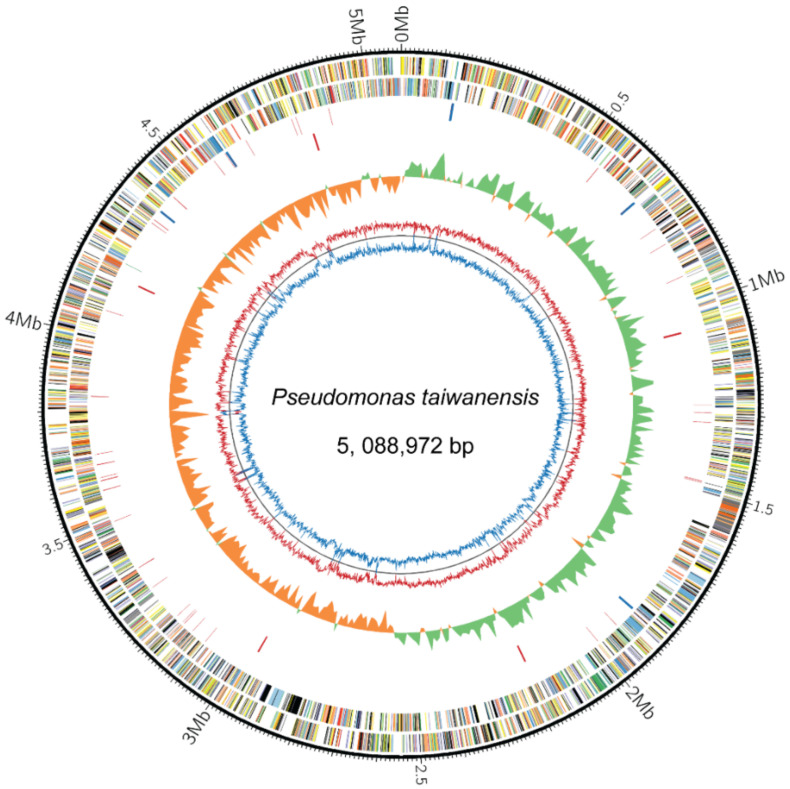
Circular representation of the *P. taiwanensis* genome (NCBI accession no. CP011858). The outermost circle shows the scale, with a resolution of 10 Kb. The genome map of *P. taiwanensis* starts with gene *dnaA*. Circles 1 and 2 show predicted coding regions color coded on the forward and reverse strands by COG assignment: red, RNA processing and modification; green, chromatin structure and dynamics; blue, energy production and conversion; purple, cell cycle control and mitosis; yellow, amino acid metabolism and transport; orange, nucleotide metabolism and transport; grey, carbohydrate metabolism and transport; dark red, coenzyme metabolism; dark green, lipid metabolism; dark blue, translation; dark purple, transcription; dark yellow, replication and repair; dark orange, cell wall/membrane/envelop biogenesis; dark grey, cell motility; light red, post-translational modification, protein turnover, chaperone functions; light green, inorganic ion transport and metabolism; light blue, secondary structure; light purple, general functional prediction only; light yellow, function unknown; light orange, signal transduction; light grey, intracellular trafficking and secretion; black, not in COGs. Circle 3 shows rRNA, tRNA, and ncRNA. Circle 4 shows two-component system *envZ* genes. Circle 5 shows GC skew in a 1000-bp window. Circles 6 and 7 show GC content (purple) and AT content (yellow) in a 1000-bp window, respectively.

The genome of *P. taiwanensis* genome was sequenced and generated via a *de novo* assembly from 454 (Roche) long reads and paired-end Illumina short reads. Using Solexa paired-end sequencing, the total output produces 3389 million paired-end total read length with an average length of 79 bases after adaptor trimming, giving approximately 666-fold coverage of the genome. In addition, the 454 sequencing produces total output of 248 million read length with an average length of 382 bases after adaptor trimming, giving approximately 48.7-fold coverage of the genome. The contigs from Solexa and 454 were organized by high-resolution restriction map (optical map) from a single strain DNA of *P. taiwanensis* (Supplementary Figure S1). The *P. taiwanensis* genome is composed of one circular chromosome of 5,088,972 base pairs (NCBI accession no. CP011858) with 62.6% GC content and annotated to encode 4477 proteins (Figure 1). *P. taiwanensis* carries 16 rRNA, 68 tRNA, and 1 ncRNA genes. In GC skew analysis [15], the chromosome of *P. taiwanensis* revealed two typical GC skew transitions, which correspond to the replication origin (*ori*) and terminus (*ter*) (Circle 5, Figure 1). The chromosomal origin of replication (*oriC*) contains a specific DnaA-binding site which is located near the *dnaA* gene [16] (25). In most prokaryotic chromosomes, the leading strand is G-rich and the lagging strand is C-rich [17]. In *P. taiwanensis*, the GC skew shows a symmetrical structure of *oriC* located opposite the terminus position (Circle 5, Figure 1) and the cumulative GC skew displays a typical mountain shape (see Figure S2). The origin and terminus axis are related to bacterial growth efficiency and replication mechanism [18]. *P. taiwanensis* has a general structure of symmetry between the leading and lagging strand, which is similar to *P. entomophila* L48, whereas *P. putida* KT2440 and *P. aeruginosa* PAO1 have asymmetrical structures [4,19,20].

In genome annotation, encoded proteins were assigned to different clusters of orthologous groups (COGs) functional classifications using the COGs database [21]. In *P. taiwanensis*, 4477 predicted proteins were assigned to different COG functional classifications of which 6.2% had unknown functions and 20.9% of coding sequences (CDS) did not hit any sequence against the COG database (see Supplementary Figure S3). The large categories of functional genes included amino acid transport and metabolism (9.3%), general function (8.2%), and transcription (7.1%) (see Supplementary Figure S3). The amino acid metabolism and transport group was also found to be the most abundant group of functional genes in the soil bacterium *Pseudomonas putida* [22,23]. This suggests that *P. taiwanensis*, which exists in the same niche as *P. putida*, may utilize amino acids as carbon and nitrogen sources from the environment. In bacterial competition, amino acid metabolic genes of *E. coli* are strongly induced during gut colonization, which affects nutrient competition among commensal intestinal bacteria [24]. The large reservoir of amino acid metabolism-related genes may also be an important factor where *P. taiwanensis* can colonize in the midgut of *Plutella xylostella* after oral infection [8]. In addition, another large group in the genome of *P. taiwanensis* is assigned to the category related to transcription, which might be required for diverse regulatory gene expression to adapt to different environments (see Supplementary Figure S3).

### 2.2. Phylogenetic Analysis of P. taiwanensis

**Figure 2:**
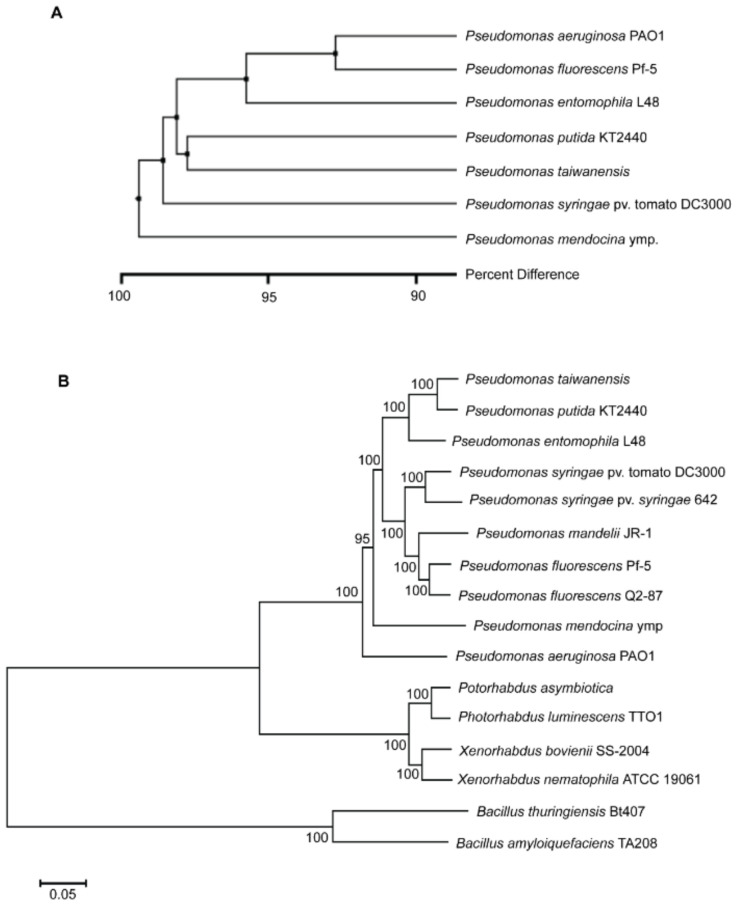
Phylogenetic analysis of *P. taiwanensis* compared with representative *Pseudomonas* species and other anti-insect and anti-microbial bacteria. (**A**) Phylogenetic analysis of the BamH1 whole-genome optical map of *P. taiwanensis* compared to in silico BamH1-digestion maps of other *Pseudomonas* species based on the unweighted-pair group method with arithmetic averages (UPGMA). (**B**) Neighbor-joining (NJ) tree analysis of several representative *Pseudomonas* species and anti-insect or anti-microbial bacteria by multilocus sequence typing (MLST) based on seven housekeeping genes (*rpoD*, *gyrB*, *acnB*, *cts*, *gap*, *pgi*, and *pfk*). The branch support of the NJ tree is calculated using a set of 1000 bootstrap replicates and the p-distance method. The unit for branch length is substitutions/site. Gram-positive bacteria *Bacilli* are used as an outgroup.

Phylogenetic analysis of *P. taiwanensis* was performed by optical restriction mapping based on the unweighted-pair group method with arithmetic averages (UPGMA) [25]. The whole genome optical BamHI-restriction map of *P. taiwanensis* was compared to those of other *Pseudomonas* species that were derived from public genomic sequences with in silico BamHI digestion (Figure 2A). The whole genome restriction map of *P. taiwanensis* displays over 95% difference compared to those of other *Pseudomonas* species. This indicates that the genomes of *Pseudomonas* genus are highly diverse. Furthermore, 16 bacteria belonging to four different genera, including plant pathogens, human pathogens, and biocontrol agents, were subjected to phylogenetic analysis based on seven housekeeping genes using multilocus sequence typing (MLST) analysis (Figure 2B). The phylogenetic tree clearly shows that bacteria can be separated into 4 different groups. The Gram-positive bacteria *Bacillus* can be distinguished from other Gram-negative bacteria. The whole genome optical BamHI-restriction map (Figure 2A) and multilocus sequence typing (MLST) analyses (Figure 2B), and the phylogenetic trees show a close relationship between *P. taiwanensis* and other *Pseudomonas* species. Phylogenomics and systematics of the genus *Pseudomonas* have been extensively studied [26,27]. The phylogenetic trees shown in Figure 2 are consistent with published phylogenetic trees. In the trees, *P. taiwanensis* is most closely related to *P. putida* KT2400 and its second closest relation is the insect pathogen *P. entomophila* L48 in the phylogeny. *P. aeruginosa*, which is an opportunistic pathogen associated with humans and other vertebrates [2], is located farther away from *P. taiwanensis* on the tree. However, *P. taiwanensis* does not have a cytotoxic effect on mouse macrophages and may not be pathogenic to mammals [8].

### 2.3. Comparison of the Genomes of P. taiwanensis, P. putida KT2400, and P. entomophila L48

Despite the close relatedness of *P. taiwanensis* to *P. putida* KT2400 and *P. entomophila* L48, the genome sizes among these three species are different. In comparison to *P. putida* KT2400 and *P. entomophila* L48, the *P. taiwanensis* genome has a smaller size that contains fewer open reading frames (4477), tRNA (69), and rRNA (16) (Supplementary Table S1). However, the *P. taiwanensis* genome contains an intact prophage with a size of 106 Kb, which is larger than those of *P. putida* KT2400 and *P. entomophila* L48.

**Figure 3:**
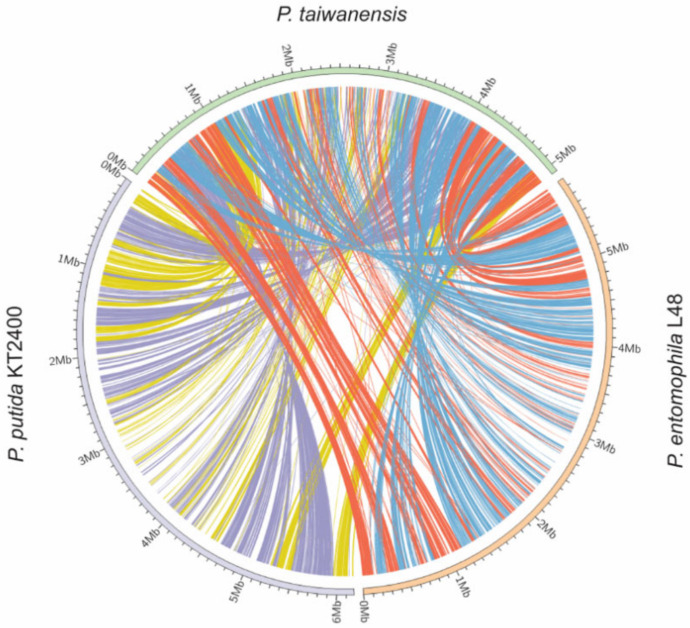
Pairwise genome comparison based on the Artemis comparison tool (ACT). Visualization of comparisons between genomes uses Circos. Blue and purple lines are reverse orientation regions between genomes. Web-based implementation of the Artemis Comparison Tool (WebACT) with default values was used.

To detect global sequence similarity, we used MUMmer to align genomes and observe homology between *P. taiwanensis* (*Y*-axis) and *P. putida* KT2400 (*X*-axis) or *P. entomophila* L48 (*X*-axis). MUMmer software revealed an X-shape alignment pattern in the dot plot in which a conserved region formed a straight line and a scattered distribution indicates less homology between two species. *P. taiwanensis* displayed less homology of large genomic regions when compared to *P. putida* KT2400 and *P. entomophila* L48 (see Figure S4). Next, we used the Artemis Comparison Tool (ACT) for pairwise genomic sequence comparison [28] and visualized the comparison of the three genomes using Circos [29]. Figure 3 shows the genome arrangement of *P. taiwanensis, P. putida* KT2400 and *P. entomophila* L48. Orange and yellow lines connect the positions of sequence similarities among *P. taiwanensis*, *P. putida* KT2400, and *P. entomophila* L48. In contrast, light blue and purple lines connect the inverted positions (Figure 3). A large rearrangement profile was detected when the *P. taiwanensis* genome was compared with those of *P. putida* KT2400 and *P. entomophila* L48. Even though *P. taiwanensis* and *P. putida* KT2400 are more closely related to each other than to *P. entomophila* according to phylogenetic analysis (Figure 2), we found that *P. taiwanensis* has a lower synteny with other Pseudomonas species (Figure 3).

### 2.4. Identification of P. taiwanensis-Specific Protein Families

**Figure 4:**
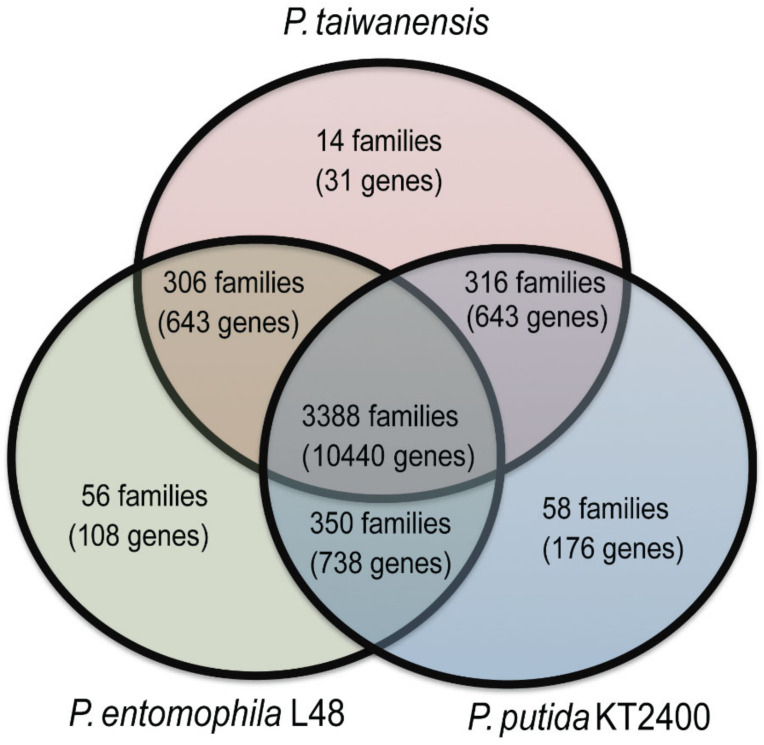
Venn diagram illustrating the distribution of gene families among three genomes, *P. taiwanensis*, *P. putida* KT2400, and *P. entomophila* L48. Homologous genes in *P. taiwanensis*, *P. putida* KT2400, and *P. entomophila* L48 were clustered into gene families. Each division of the Venn diagram shows orthoMCL groups and total number of clustered genes.

We used the orthoMCL [30] to define homologous proteins and clustered orthologs among *P. taiwanensis*, *P. putida* KT2400, and *P. entomophila* L48 genomes. A total of 4488 families were identified, of which 3388 families containing 10,440 protein-coding genes were common among all three genomes (Figure 4). Thirty-one protein-coding genes belonging to 14 families were found only in *P. taiwanensis,* 176 protein-coding genes belonging to 58 families were found only in *P. putida* KT2400, and 108 coding-genes belonging to 56 families were found only in *P. entomophila* L48. *P. taiwanensis* specific proteins are shown in Supplementary Table S2.

Among the 14 gene families specific to *P. taiwanensis*, several families contain genes encoding pathogenesis-related proteins, including oxidoreductase, GntR transcriptional regulator, LuxR transcriptional regulator, acyl-CoA dehydrogenase, and amidohydrolase. The oxidoreductase enzymes play roles in antioxidative response which is important for bacterial pathogens to successfully overcome the defense systems of the host [31]. A high antioxidant response was shown for *P. taiwanensis* [8], suggesting that oxidoreductase family enzymes might participate in pathogenic activity. GntR family transcriptional regulators include more than 1300 members and function in regulating gene expression in response to environmental stresses [32]. LuxR family proteins regulate a variety of genes involved in the production of virulence factors, biosynthesis of antibiotics, formation of biofilm, extracellular protease, extracellular polysaccharide, bioluminescence, swarming, mobility, plasmid conjugal transfer, and nodulation [33]. LuxR transcriptional regulator is an essential player for quorum sensing. LuxR can be activated by acyl-homoserine lactone (AHL) that is synthesized by LuxI [33]. However, some LuxI/LuxR family members appeared to have been inherited from other bacteria through horizontal transfer [34]. Horizontal transfer of individual LuxR may provide a specific role in the regulatory cassette, for example, the two LuxR family members CarR and ExpR can active different sets of genes in *Erwinia carotovora* subsp. *Carotovora* [35]. Therefore, specific LuxR-like homologs may regulate downstream gene sets for anti-bacterial or anti-insect activity in *P. taiwanensis*. The acyl-CoA dehydrogenase family catalyzes the oxidation of branched-chain amino acids into the fatty acid precursors and lipopeptide, macrolide antibiotic synthesis [36]. The medium/long-chain fatty acyl-CoA dehydrogenase plays a role in carbon starvation-stress [37]. *P. taiwanensis* induced higher toxicity against rice pathogen *Xoo* under iron-limited minimal medium compared to other *Pseudomonas* [11]. Acyl-CoA dehydrogenase may be involved in the virulence factor production by *P. taiwanensis* when it is grown on nutrient-limited minimal medium. Another unique gene is amidohydrolase which belongs to the amidohydrolase superfamily and catalyzes the degradation of xenobiotics [38]. Amidohydrolase may participate in the defense mechanism in the communication between *P. taiwanensis* and competitor or host.

### 2.5. Identification of Genes Involved in Anti-Xoo Via Tn5 Transposon Mutagenesis

In our prior studies, we found that *P. taiwanensis* utilized iron-chelated pyoverdine to inhibit the growth of rice pathogen *Xoo* [11]. Several Tn*5*-tagged mutants of *P. taiwanensis* that are defective in the biosynthesis and secretion of pyoverdine were isolated and characterized [11]. To gain a more comprehensive picture of the mechanism of anti-*Xoo* activity in *P. taiwanensis*, we performed a large-scale screening of the Tn*5* mutagenisis library for additional mutants with reduced anti-*Xoo* activity. Among 11,646 Tn*5*-inserted mutants screened, 913 displayed decreased anti-*Xoo* activities compared to the wild type and 19 had greater anti-*Xoo* activities (Supplementary Tables S3 and S4). To determine Tn*5* transposon insertion sites, thermal asymmetric interlaced PCR (TAIL-PCR) was used to detect the flanking sequence of the transposon. The TAIL-PCR products were sequenced and subjected to BLAST search against the CDS of *P. taiwanensis* (NCBI accession no. CP011858) and the *Pseudomonas* database.

**Figure 5:**
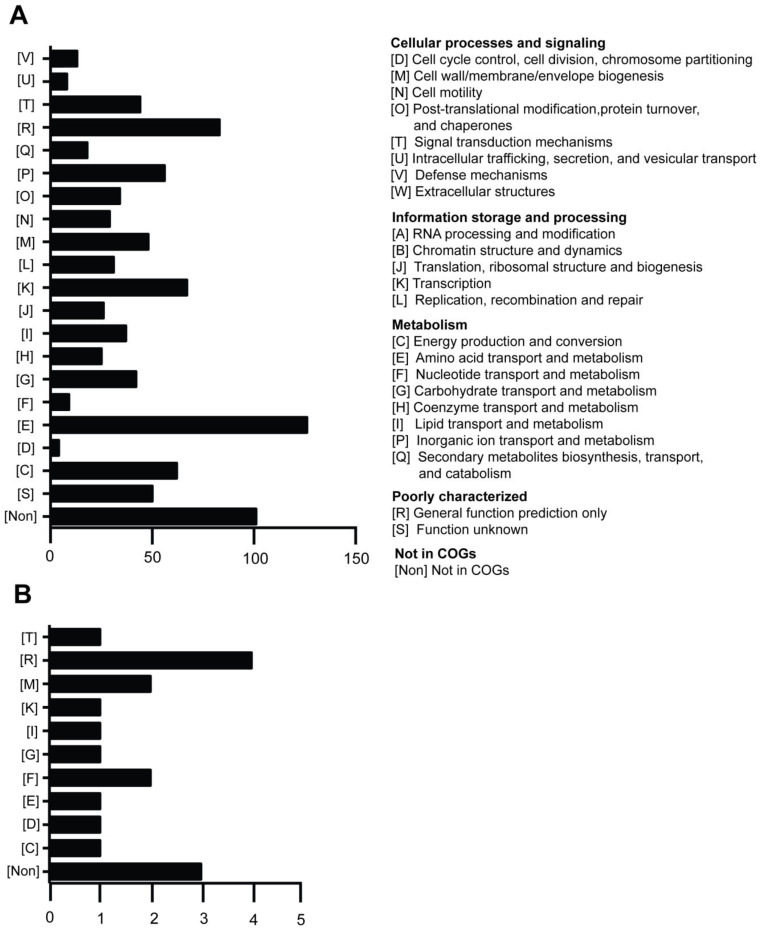
Clusters of orthologous groups (COG) analysis of *P. taiwanensis* involved in anti-*Xoo* activity. (**A**) A total of 913 genes were determined to have decreased anti-*Xoo* activity among 11,646 Tn*5*-inserted mutants (**B**) 19 genes were determined to have increased toxicity against *Xoo*. All genes were annotated by COG database. The number on the X axes represent the number of genes.

The genes of the 913 Tn*5*-inserted mutants were annotated by the COG database. These genes were found to be involved in metabolism, information storage and processing, cellular processes and signaling (Figure 5A and Supplementary Table S3). Many Tn*5*-inserted genes play a critical role in cell growth in producing energy. The largest group of genes was not identified in the COG database. A number of genes were categorized as virulence-related genes based on the anti-*Xoo* activity. We confirmed that these virulence-related mutants have a single-copy Tn*5* insertion by Southern blot analysis (see Supplementary Figure S5). Our previous studies showed that the iron-chelator pyoverdine is a key virulence factor against rice pathogen *Xoo* [11]. In addition, we showed that *pvdL* and *pvdE* mutants did not produce mature pyoverdine (*m*/*z* 1044) and have no toxicity against *Xoo*. The *pvdL* gene encodes non-ribosomal peptide synthetase (NRPS) which is involved in the biosynthesis of pyoverdine chromophore [39]. PvdE, an inner membrane transporter, is responsible for the translocation of pyoverdine precursor into the periplasm to form mature pyoverdine [40]. From the 913 Tn*5*-inserted mutants, we collected genes that have been shown to synthesize and regulate iron-chelator pyoverdine (Table 1). There are four pyoverdine-deficient mutant strains, which encode proteins required for mature pyoverdine synthesis (Table 1). Besides *pvdL* and *pvdE*, we also found other pyoverdine synthesis genes *pvdI* and *pvdQ*. The *pvdI* gene encodes NRPS and is involved in the biosynthesis amino acids of pyoverdine [40]. The *pvdQ* gene encodes acyl-homoserine lactone acylase which is required for pyoverdine biosynthesis [41]. Pyoverdine can inhibit and kill *Xoo* in *P. taiwanensis* through iron competition, which can be secreted through an unknown mechanism involving T6SS [11].

On the other hand, we found that 19 Tn*5*-inserted mutants displayed higher toxicity against *Xoo* than the wild type (see Supplementary Table S4). These genes are involved in metabolism (energy production and conversion, 1; amino acid transport and metabolism, 1; nucleotide transport and metabolism, 2; carbohydrate transport and metabolism, 1; lipid transport and metabolism, 1, information storage and processing (Transcription, 1); cellular processes and signaling (cell cycle control, cell division, chromosome partitioning, 4; cell wall/membrane/envelop biogenesis, 48; cell motility, 29; posttranslational modification, protein turnover, chaperones, 3; signal transduction mechanisms, 1), poorly characterized (general function prediction only, 4) and not in COGs (not in COGs, 3) (Figure 5B and see Supplementary Table S4).

### 2.6. Positive Regulation of Pyoverdine by Two-Component System EnvZ/OmpR

**Figure 6:**
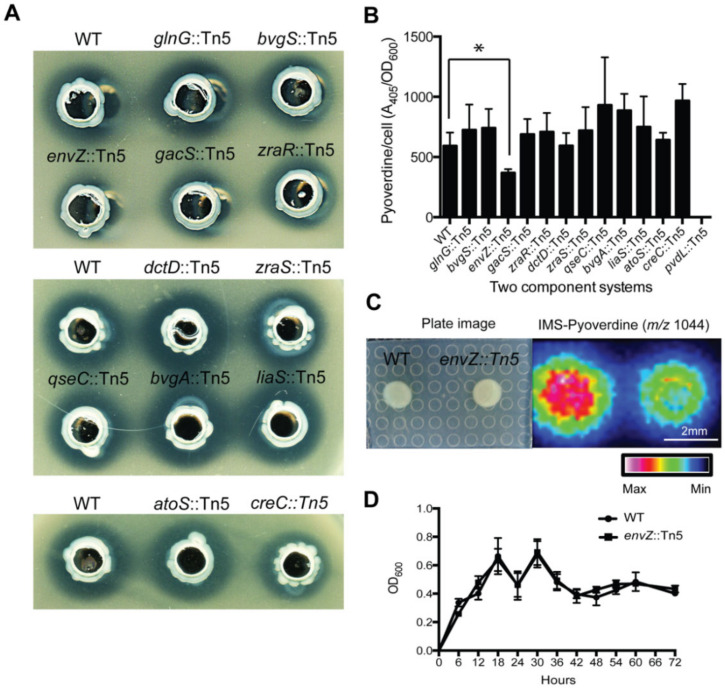
Pyoverdine secreted by type VI secretion system and regulated by EnvZ/OmpR. (**A**) We compared the capabilities of the wild type and 12 TCS mutants (*bvgS*, *bvgA*, *envZ*, *atoS*, *gacS*, *zraR*, *glnG*, *dctD*, *gseC*, *liaS*, *creC*, *zraS*) of *P. taiwanensis* against rice pathogen *Xanthomonas oryzae* pv. *oryzae* (*Xoo*). (**B**) Quantification of extracellular mature pyoverdine was achieved by measuring fluorescence at excitation 405 nm and emission 460 nm. Pyoverdine values were normalized against the cell optical densities (Ex405, Em 460/OD_600_). Among the mutants being examined, only *envZ*::Tn*5* mutant showed significantly lower pyoverdine production. * *p* < 0.05. (**C**) MALDI-IMS analysis reveals secreted pyoverdine concentration around wild-type and *envZ* mutant colonies. (**D**) Growth curves of wild type and *envZ* mutant were measured by optical density at 600 nm. Bacteria were grown for 24 h on iron-limited agar plates. Intensity gradients for pyoverdine by color histograms (maximum, white; minimum, black). Scale bar, 2 mm.

Among the 913 Tn*5*-inserted mutants, we also identified genes that are potentially involved in regulating the production of pyoverdine (Table 1). Two component systems (TCSs) are widely distributed in prokaryotes and perform signal transduction in response to environmental stimulations by regulating downstream gene expression [42]. Moreover, TCSs exhibit effects on virulence gene expression in prokaryotes, such as PhoP-PhoQ regulating virulence in *Salmonella,* and BvgA-BvgS being a dominant regulator of virulence in *Bordetella pertussis* [43]. In *P. aeruginosa* and *P. syringae*, GacA-GacS plays an important role in pathogenesis in plants and animals [44]. GacA-GacS is also a broad regulator of secondary metabolite synthesis in *P. fluorescens* [45]. In a mutant library of *P. taiwanensis,* we compared anti-*Xoo* activities in mutants of 12 TCS (*bvgS*, *bvgA*, *envZ*, *atoS*, *gacS*, *zraR*, *glnG*, *dctD*, *gseC*, *liaS*, *creC*, *zraS*) under iron-limited conditions (Figure 6A). TCS mutants *envZ*::Tn*5*, *gacS*::Tn*5*, *zraR*::Tn*5* and *zraS*::Tn*5*, *creC*::*Tn5* showed lower toxicity against *Xoo* compared to other TCS mutants (Figure 6A). However, only *envZ*::Tn*5* mutant showed significantly lower pyoverdine production (Figure 6B). Furthermore, by matrix-assisted laser desorption/ionization-imaging mass spectrometry (MALDI-IMS) detection, *envZ*::Tn*5* mutant secreted a lower level of pyoverdine (*m/z* 1044) compared to wild type (Figure 6C). *EnvZ*::Tn*5* mutant did not affect growth compared to wild type under iron-limited conditions (Figure 6D). The copy numbers of Tn*5* insertion in each TCS mutant were determined by Southern blot analysis. The results showed that except for *lias*::Tn*5* mutant, all the TCS mutants contained a single-copy Tn*5* insertion (see Supplementary Figures S5 and S6).

EnvZ/OmpR has been shown to display functional diversity across a wide range of bacteria. In *E.coli*, EnvZ/OmpR plays a major role in mediating osmotic and ironic balance by regulating signal transduction pathways that affect more than 100 genes in metabolism and motility [46]. In *Salmonella typhimurium*, EnvZ/OmpR can affect gene expression of the type III secretion system under low osmolarity, acidic pH and the absence of Ca^2+^ [47]. On the other hand, in *Shewanella oneidensis*, EnvZ/OmpR displays response to alkaline environments and affects cell motility [48]. In pathogenesis, OmpR, phosphorylated by EnvZ, can regulate a variety of virulence genes in the pathogens *Shigella flexneri*, *Yersinia enterocolitica, Salmonella typhi*, and *S. typhimurium* [49]. In this study, we found that Tn*5*-transposon insertion mutant of envZ affect extracellular level of pyoverdine in an iron-limited environment (Figure 6).

### 2.7. Negative Control of Pyoverdine Production by RpoS

**Figure 7:**
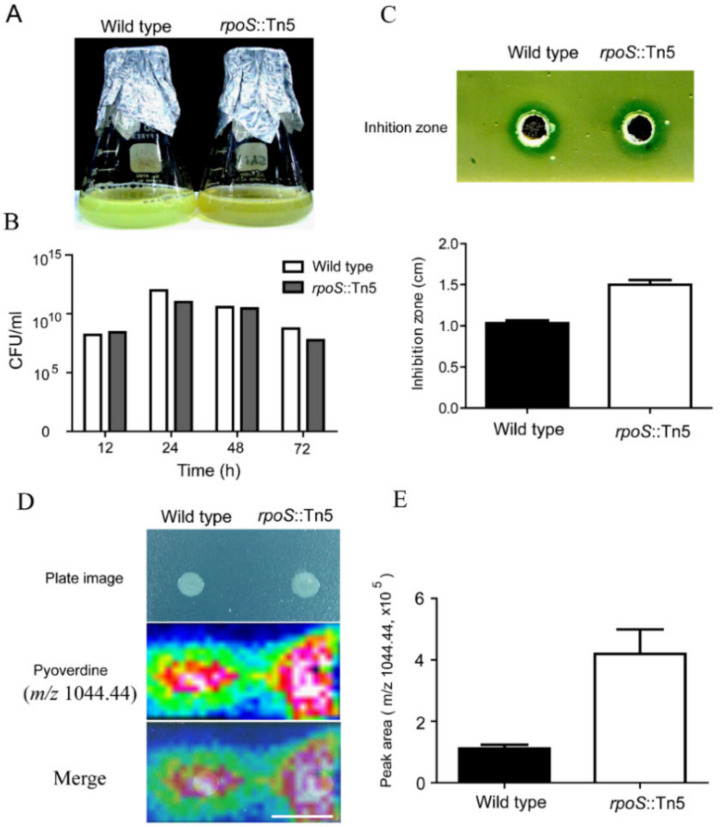
Negative regulation of pyoverdine by RpoS. (**A**) Wild type and *rpoS* mutant were incubated for 72 h at 28 °C with 200 rpm in iron-limited medium, and (**B**) numbers of CFU of wild type and *rpoS* mutant were measured during 72 h incubation. (**C**) In antagonistic assay, total broth of wild type and mutant after 72 h incubation were placed into the hole of *Xoo*-containg 1/2 agar plate, and then the inhibition zone was measured (cm). (**D**) MALDI-IMS of pyoverdine from wild type and *rpoS* mutant on the surface of an iron-limited agar plate after 72 h incubation. (**E**) Quantification of pyoverdine from culture supernatant of wild type and *rpoS* mutant after 72 h incubation using LTQ-Orbitrap mass spectrometer. Intensity gradients for pyoverdine as color histograms (maximum, white; minimum, black). Scale bar, 2 mm.

The stationary phase sigma factor, RpoS, is a global stress response regulator. We identified an *rpoS* mutant of *P. taiwanensis* that showed increased pyoverdine production in iron-limited medium compared to the wild type (Figure 7). Cultures of the *rpoS* mutant strain exhibited deep green color under iron-limited medium compared to light green color in the wild type after three days of flask incubation (Figure 7A), and *rpoS* mutation did not affect cell growth (Figure 7B). The deep green color in the *rpoS* mutant cultures likely resulted from the amount of florescent pigment pyoverdine accumulating in the medium. In antagonistic assay, the *rpoS* mutant had a larger inhibition zone than the wild type toward *Xoo* (Figure 7C). Furthermore, we utilized IMS to directly detect the concentration of pyoverdine on agar plates and determine whether *rpoS* affects pyoverdine production. IMS data showed that the amount of pyoverdine in the *rpoS* mutant was more than that secreted by the wild type (Figure 7D). The quantification of pyoverdine showed that the *rpoS* mutant had a 2–3 fold higher concentration of pyoverdine in iron-limited supernatant compared to that of wild type by LC-MS (Figure 7E). Southern blot analysis confirmed that *rpoS*::Tn*5* mutant only carries a signal copy of Tn*5* (see Supplementary Figure S7).

Our results suggest that pyoverdine is negatively regulated by RpoS in *P. taiwanensis* in iron-limited medium. RpoS has been shown to negatively regulate pyoverdine production in *P. aeruginosa* [50,51], but has has no effect on siderphore production in *P. putida* [52]. In *P. aeruginosa,* RpoS positively regulates the expression of *fur* gene, which encodes a transcriptional repressor for pyoverdine biosynthesis regulator PvdS, when the cells are grown in iron-rich medium [53]. These results suggested that iron homeostasis plays a role in regulating pyoverdine production in *Pseudomonas*.

## 3. Materials and Methods

### 3.1. Bacterial Growth Conditions and Antagonistic Assay

*Pseudomonas taiwanensis* sp. nov. CMS^T^ (= BCRC 17751^T^ = DSM 21245^T^) was used in this study [11]. Antagonistic assay of *P. taiwanensis* against *Xoo* was conducted on 1/2 trypticase soy (TSB) agar plates (BD Biosciences, San Jose, CA, USA) at 28 °C. *P. taiwanensis* was grown in 100 mL of iron-limited liquid medium (M9 minimal medium supplemented with 1% casamino acids, 1 mM MgSO_4_, and 0.5% glycerol) in a 500 mL flask at 28 °C and 200 rpm with shaking for 24 h. *Xoo was* cultured in 1/2 TSB medium at 28 °C for 1 day. *Xoo* was mixed with melted 1/2 TSB agar medium before being poured into an empty plate for competition assay. For bioassay, bacteria culture broth (10^9^ CFU/mL) or filtered (0.22 µm) supernatant was injected (50 µL) into the hole in the *Xoo*-mixed 1/2 TSB agar plate until the inhibition zones had been characterized. The bacteria pellets were washed three times with PBS at 4 °C and resuspended in PBS. Cell density and cell viability were determined using optical density at OD_600_ and by counting CFU/mL.

### 3.2. DNA Extraction, Genome Sequencing and Assembly

*P. taiwanensis* was grown overnight in LB broth. The Qiagen RNA/DNA mini kit (Qiagen, Hilden, Germany) was used for extraction of genomic DNA. DNA purity and concentration were determined by a nanodrop spectrophotometer and agarose DNA electrophoresis.

The genomic DNA of *P. taiwanensis* BCRC 17,751 was sequenced using the Roche 454 GS FLS and Illumina GAII performed at the High Throughput Genomics Core Facility of the Biodiversity Research Center in Academic Sinica. The DNA reads of Illumina were used to generate 120 bp pair-end read lengths, and the Roche 454 (Roche, Basel, Switzerland) was used to generate 300–400 bp read lengths. The paired-end raw reads of Illumina were trimmed to remove linkers and adaptors and further assembled by CLC Genomic Workbench (4.0.2). Trimming of adaptors and primers of Roche 454 lone reads was performed by Newbler (2.3). The contigs of Illumina and Roche 454 were oriented and assembled based on the whole genome optical BamHI-restriction map of *P. taiwanensis* (OpGen Inc., Gaithersburg, MD, USA) to construct one circular genome (Figure S1). Assembly of a BamHI-restriction map was performed by MapSolver software (OpGen). Optical mapping service was performed at Yourgene BioScience Co. (New Taipei City, Taiwan). The gaps in the genome were filled and closed by Sanger sequencing. Whole genome sequence of *P. taiwanensis* was submitted to NCBI Genbank (NCBI accession no. CP011858).

### 3.3. Annotation

The *P. taiwanensis* open reading frames (ORFs) were predicted and annotated using the NCBI prokaryotic genome annotation pipeline [54]. Annotation data of *P. taiwanensis* is deposited in NCBI (NCBI accession no. CP011858). The prophage elements were identified via PHAge Search Tool (PHAST) [55].

### 3.4. Phylogenetic Analysis

Phylogenetic trees were constructed based on an optical map and multi-locus sequence typing (MLST). A whole genome-wide phylogenetic tree was built based on the whole genome optical BamHI-restriction map data of *P. taiwanensis* compared with in silico BamHI-restriction map data of other *Pseudomonas* species using the unweighted pair group method with arithmetic mean (UPGMA). The whole genome-wide phylogenetic tree was generated in MapSolver^TM^ from optical restriction map data. The MLST phylogenetic tree analysis of *Pseudomonas* species and other biocontrol agents was built by means of the seven housekeeping genes amino acid sequence, *rpoD*, *gyrB*, *acnB, cts*, *gap*, *pgi,* and *pfk.* A neighbor-joining tree based on the amino acid sequences of seven concatenated housekeeping genes was built by using MEGA 4.0 with 1000 bootstrap replicates.

### 3.5. Whole-Genome Sequence Comparison

Whole-genome DNA alignments were generated in two ways. First, the MUMmer program was used for pair-wise alignment of two entire bacterial genomes [56]. Second, WebACT was used to generate pair-wise alignments of three entire bacterial genomes [57]. WebACT comparison data among the three genomes were visualized by the Circos software package [29].

### 3.6. Gene Family Comparison

All predicted protein sequences of *P. taiwanensis*, *P putida* KT2400, and *P. entomophila* L48 were compared to each other using BLASTP with E-value cut-off of 10^−5^. The Markov cluster (MCL) algorithm was used to identify specific gene families by clustering the BLASTP results, which can be separated into different groups based on homology of proteins, with an inflation parameter of 2.0 [58].

### 3.7. Identification of Replication Origin and Terminator

The position of the genomic replication origin was determined based on initiator gene *dnaA* and GC skew [59]. GC skew can analyze the terminator region of genome. The GC skew was calculated by (C − G)/(C + G) using a 1000 bp window size. Because G and T are enriched in the leading strand in most bacterial genomes, the GC skew profile displays asymmetry in nucleotide compositions between leading and lagging strands, which were caused by replication starting point in the different direction of leading and lagging strands.

### 3.8. Tn5 Mutant Library

We made Tn*5* insertion mutants using an EZ-Tn*5* transposon mutagenesis kit <KAN-2> (Epicentre). To determine the Tn*5* transposon insertion site, thermal asymmetric interlaced PCR (TAIL-PCR) was used to detect the sequences flanking the transposon. The tail-PCR products were sequenced and subjected to BLAST search against the *P. taiwanensis* database and *Pseudomonas* database. To screen the Tn*5* mutant library, we utilized a *P. taiwanensis* mutagenesis library to incubate with *Xoo* to find pathogenicity-related genes.

### 3.9. Pyoverdine Detection

Pyoverdine was measured from culture supernatants using fluorescence spectrometer (Synergy MX, BioTek, Winooski, VT, USA) detection at excitation wavelength 405 nm and emission wavelength 460 nm [11,60]. Pyoverdine level was normalized by the cell density (OD_600_) of cell culture. Cell density was detected at 600 nm in a microplate spectrophotometer (PowerWave XS, BioTek) after washing twice in PBS.

### 3.10. MALDI-IMS

MALDI-IMS allows us to detect pyoverdine (*m/z* 1044) on the surface of the agar plate, as reported in a previous study [11]. The ion distribution of pyoverdine on the surface of iron-limited agar plates revealed a difference in densities of pyoverdine between wild type and mutants of *P. taiwanensis*. The iron-limited agar samples with bacterial colonies were excised and placed on glass slides, and then covered with a thin layer of universal MALDI matrix (Sigma-Aldrich). Samples were detected in positive reflectron ion mode, and screened at 200 μm laser intervals with the acquisition mass range set at 1000–1500 Da. The standard peptide calibration mixture (Peptide Calibration Standard 206,195, Bruker, 1000–3200 Da) and universal MALDI matrix were used to calibrate and test the MALDI-TOF mass spectrometer. The IMS data were analyzed using Fleximaging 3.0 software (Bruker, Billerica, MA, USA). The intensity of the molecules is presented as gradient colors in the figures.

### 3.11. Southern Blot

Tn*5* insertion copy number was checked by Southern blot hybridization. EZ-Tn*5* Tnp transposon contains a kanamycin selectable marker. The coding sequence of the kanamycin resistance gene was used as a probe to detect the Tn*5* insertion copy number. Genomic DNA of wild type and Tn*5*-inserted-mutants were digested with EagI and hybridized with a DIG-labeled PCR probe. After hybridization, the signals were visualized using a DIG Luminescent Detection Kit (Roche).

## 4. Conclusions

**Figure 8:**
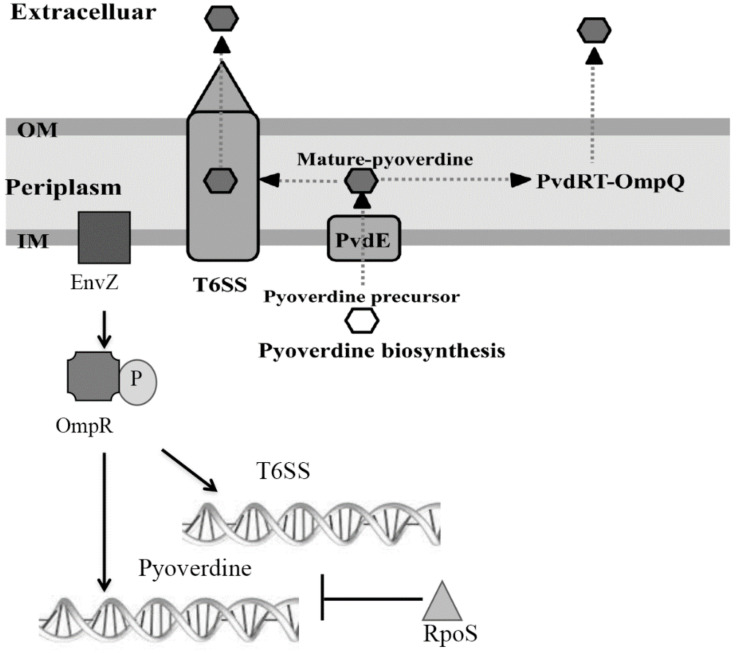
Proposed scheme for synthesis, secretion and regulation of pyoverdine.Pyoverdine precursors are synthesized in the cytoplasm and secreted into the periplasm by the inner membrane transporter PvdE, and processed into mature pyoverdine in the periplasmic space. T6SS is involved in the secretion of pyoverdine via an unknown mechanism. EnvZ/OmpR positively regulates pyoverdine production, whereas the sigma factor RpoS negatively affects pyoverdine production.

In this work, we have provided a global view of the molecular basis of *P. taiwanensis* as a biocontrol bacterium (Figure 8). Antagonistic assays of mutants revealed that the anti-*Xoo* activity of *P. taiwanensis* involves many genes that play distinct roles according to their characteristics. For induction of toxicity against *Xoo*, *P. taiwanensis* requires iron-limited conditions which activate iron-chelator pyoverdine synthesis genes and a specific EnvZ/OmpR two-component signal transduction pathway. In contrast, we found that the sigma factor RpoS negatively affects pyoverdine production. In a number of Gram-negative bacteria, EnvZ/OmpR is known to regulate osmotic stress response. However, this is the first study to show that EnvZ/OmpR plays a major role in regulating a network of downstream genes under iron-limited stress after mid-stationary phase growth of *P. taiwanensis*. EnvZ/OmpR signal transduction can be activated under iron-limited stress. In contrast, in *envZ* mutant of *P. taiwanensis*, many virulence factors show a lower level of expression in nutrient-rich conditions. The finding that the extracellular level of pyoverdine is affected Tn*5*-transposon inserted mutant of *envZ* in an iron-limited environment offers us an opportunity to examine how EnvZ/OmpR senses the iron signal to trigger signaling steps to regulate downstream genes. In the future, we will perform transcriptomic analyses of the wild type and Tn*5*-transposon tagged *envZ* mutant in iron-rich and iron-limited environments to address the role of iron in EnvZ/OmpR transduction and pyoverdine production. The results may provide information to improve the biocontrol capability of *P. taiwanensis*.

## Figures and Tables

**Table 1:** Genes involved in synthesis, secretion and regulation of pyoverdine in *P. taiwanensis.*

Gene Name	Gene Product	Function	NCBI CDS No.	Reference
pyoverdine synthesis				
*pvdI (lgrC)*	Non-ribosomal peptide synthetase	predicted to synthesis residues serine and lysine	GQ77_12920	This study
*pvdL (lgrB)*	Non-ribosomal peptide synthetase	synthesis of the pyoverdine chromophore	GQ77_07720	
*pvdQ*	Acyl-homoserine lactone acylase pvdQ	Catalyzes the deacylation of acyl-homoserine lactone	GQ77_10045	This study
*pvdE (syrD)*	Pyoverdine ABC transporter	pyoverdine translocation and maturation	GQ77_12890	[40]
Involved in pyoverdine secretion				
*clpV1*	Type VI secretion ATPases with chaperone activity, ATP-binding subunit	Required for secretion of hcp1 probably by providing the energy source for its translocation	GQ77_17045	[40]
*icmF*	ImcF domain-containing protein	VI_IcmF: type VI secretion protein IcmF	GQ77_17050	[40]
*tssC*	Type VI secretion protein EvpB	Unknown	GQ77_17105	[40]
Positive regulation of pyoverdine				
*envz*	Osmolarity sensor protein envZ	Signal transduction histidine kinase, two-component signal transduction system	GQ77_09865	This study
Negative regulation of pyoverdine				
*rpoS*	RNA polymerase sigma factor rpoS	DNA-directed RNA polymerase, sigma subunit (sigma70/sigma32)	GQ77_05495	This study

## Supplementary Materials

Supplementary Materials can be found at https://www.mdpi.com/1422-0067/21/22/8639/s1. Figure S1. Assembly scheme and annotation pipeline. Figure S2. Cumulative and normal GC-skew analysis of *P. taiwanensis*. Figure S3. Clusters of orthologous groups functional classification of predicted proteins encoded in the *P. taiwanensis* genome. Figure S4. Whole genome alignment between *P. taiwanensis* (*y*-axis) and *P. entomophila* L48 (*x*-axis) or *P. putida* KT2400 (*x*-axis). Figure S5. Southern blot analysis of EagI-digested genomic DNA of WT and virulence-related Tn*5*-inserted mutants. Figure S6. Southern blot analysis of EagI-digested genomic DNA of WT and Tn*5*-inserted mutants of two component systems. Figure S7. Southern blot analysis of EagI-digested genomic DNA of WT and Tn*5*-inserted mutants of sigma factor *rpoS. Tn5*-inserted mutant of *rpoS* was identified as having a single-copy Tn*5* insertion. NCBI CDS accession number: *rpoS* (GQ77_05495). Table S1. General features of genomes of *P. taiwanensis* and its close-related species *P. entomophila* L48 and *P. putida* KT2400. Table S2. Specific gene families of *P. taiwanensis* compared to *P. putida* KT2400 and *P. entomophila* L48. Table S3. List of Tn*5*-inserted mutants display decreased anti-*Xoo* activity compared to wild type. Table S4. List of Tn*5*-inserted mutants displays higher toxicity against *Xoo* than wild type.

## Abbreviations

*P. taiwanensis*	*Pseudomonas taiwanensis*
*XOO*	*Xanthomonas oryzae* pv.*oryzae (Xoo)*
MALDI	matrix-assisted laser desorption/ionization
IMS	imaging mass spectrometry
