# Supplementary material for: Whole Genome Sequencing and Tn5-Insertion Mutagenesis of Pseudomonas taiwanensis CMS to Probe Its Antagonistic Activity Against Rice Bacterial Blight Disease
Source: Int J Mol Sci. 2020 Nov 16;21(22):8639. doi: 10.3390/ijms21228639 (PMC7696974; doi:10.3390/ijms21228639)
Supplement: Supplementary file 1 [file ijms-21-08639-s001.zip › New Supplemental FIGURE S1-S7.pdf]

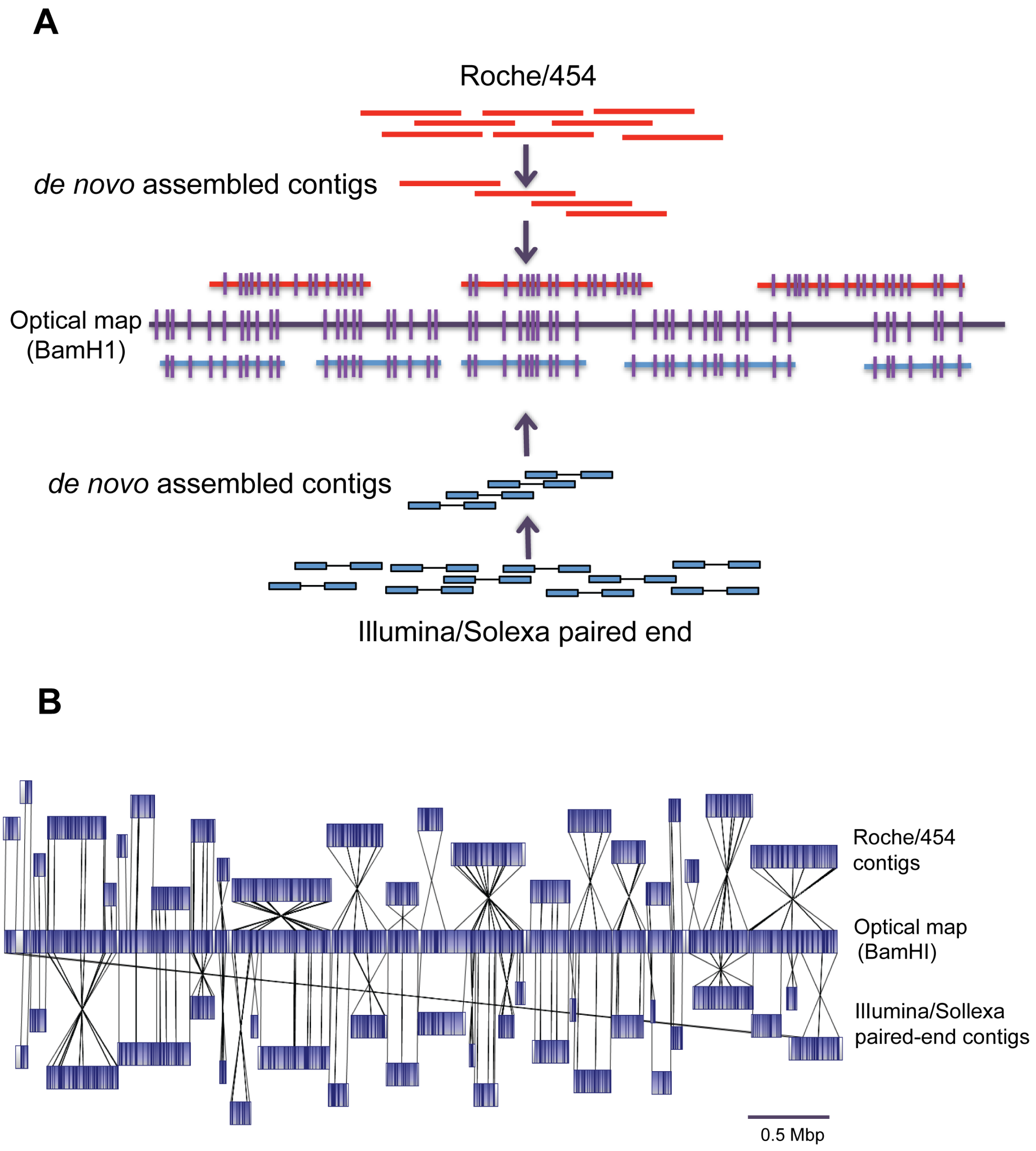

FIGURE S1. Assembly scheme and annotation pipeline. (A) Step 1: De novo assembly of *P. taiwanensis* genome with Roche/454 and Illumina/Solexa reads, respectively. Step 2: Scaffolding and orientation of 454 and Solexa contigs using optical BamH1 restriction maps. Step 3: Gaps were filled by Sanger sequencing. (B) Optical-BamH1 restriction map comprised 23 contigs from 454 and 28 contigs from Sollexa.

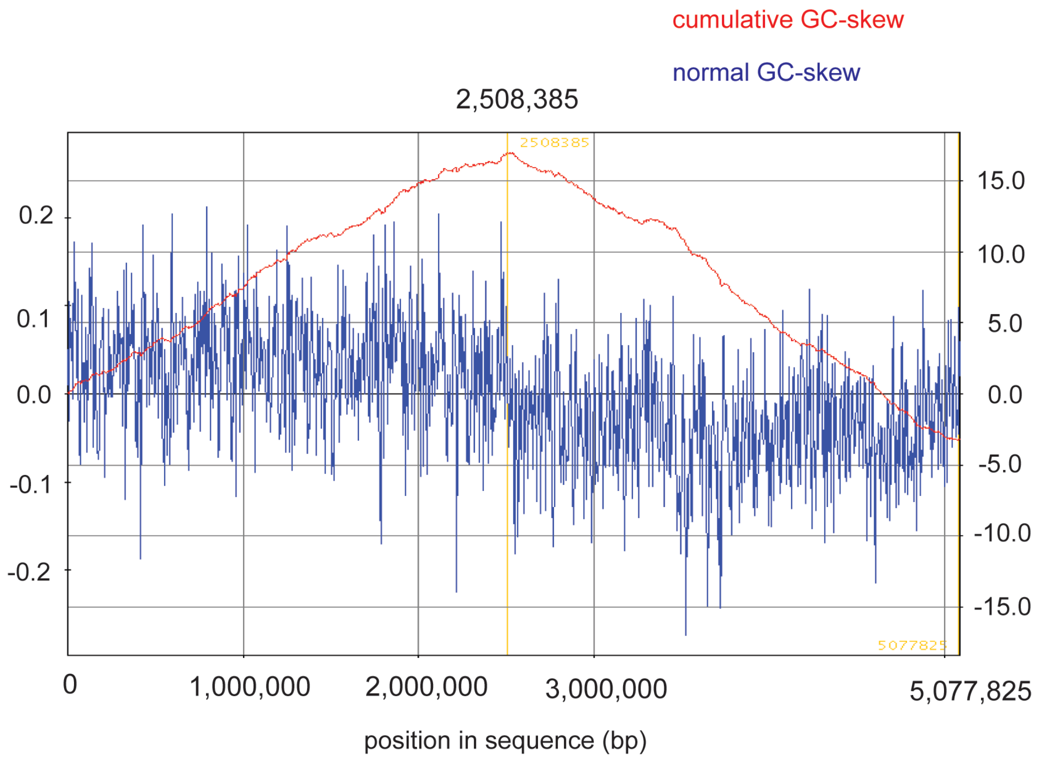

FIGURE S2. Cumulative and normal GC-skew analysis of the *P. taiwanensis*. The predicted origin of replication (*oriC*) was marked at position +1. The predicted terminus (ter) position is located at 2,508,385 bp. This plot exhibits a typical mountain shape, which has a symmetric structure with the *oriC* located opposite the terminus region.

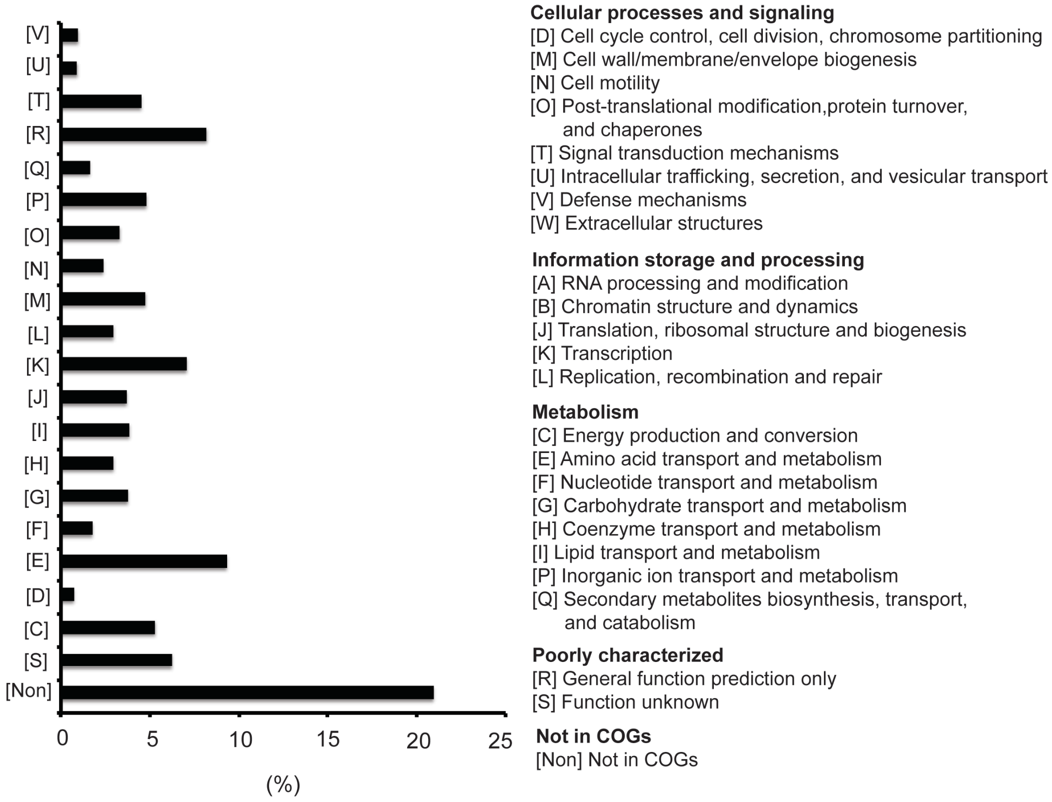

FIGURE S3. Clusters of orthologous groups (COG) functional classification of predicted proteins encoded in *P. taiwanensis* genome. The X-axis displays the percentage of a specific COG category in the distribution of *P. taiwanensis* genome. The Y-axis displays COG categories.

A

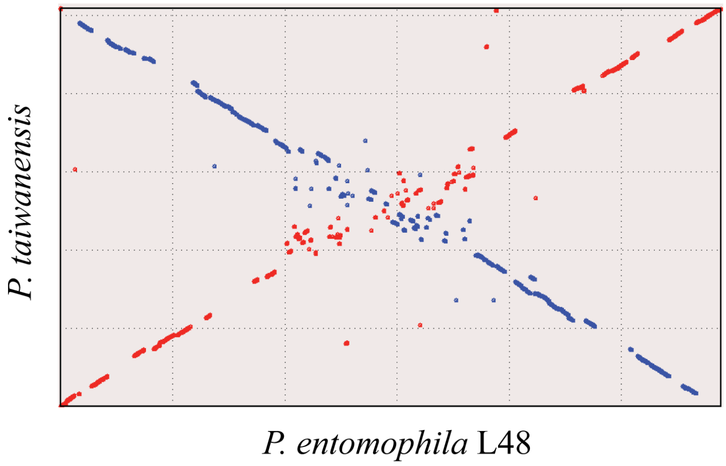

B

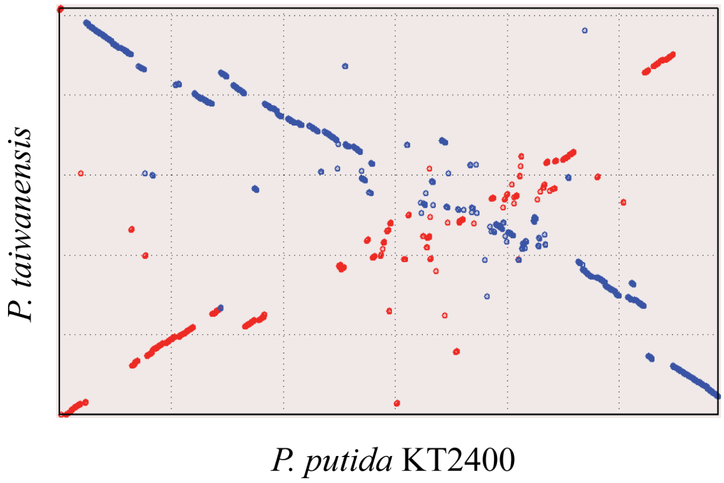

FIGURE S4. Whole genome alignment between the nucleotide positions of *P. taiwanensis* (y-axis) and *P. entomophila* L48 (x-axis) or *P. putida* KT2400 (x-axis). Liner and scattered regions indicate more conserved and less conserved regions, respectively. Whole genome comparison was performed using the MUMmer alignment software with default values.

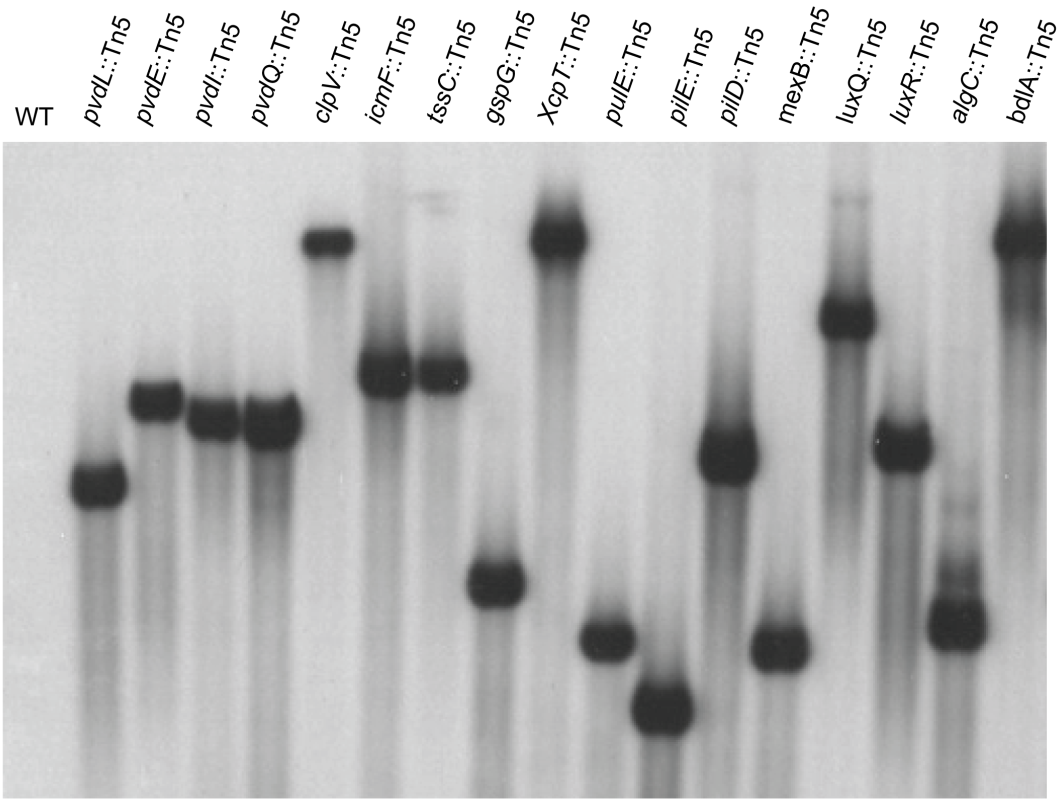

FIGURE S5. Southern blot analysis of EagI-digested genomic DNA of WT and virulence-related Tn5-inserted mutants. Mature pyoverdine synthesis-relative (*pvdL*, *pvdE*, *pvdI*, pvdQ), Type VI secretion system components (*clpV*, *icmF*, *tssC*), Type II secretion system components (*gspG*, *XcpT*, *pulE*), type IV pili biogenesis protein (*pilE*), type IV prepilin peptidase (*pilD*), multidrug exporter (*mexB*), lux system (*luxQ*, *luxR*) and biofilm formation (*algC*, *bdlA*) mutants were identified as a single-copy insertion of Tn5. Wild-type *P. taiwanensis* was used as a negative control. NCBI CDS accession number: *pvdL* (GQ77_07720), *pvdE* (GQ77_12890), pvdI (GQ77_12920), *pvdQ* (GQ77_10045), *clpV* (GQ77_17045), *icmF* (GQ77_17050), *tssC* (GQ77_17105), *gspG* (GQ77_10560), *xcpT* (GQ77_10575), *pulE* (GQ77_00900), *pilE* (GQ77_19615), *pilD* (GQ77_19525), *mexB* (GQ77_10680), *luxQ* (GQ77_08860), *luxR* (GQ77_09335), *algC* (GQ77_15925), *bdlA* (GQ77_11790).

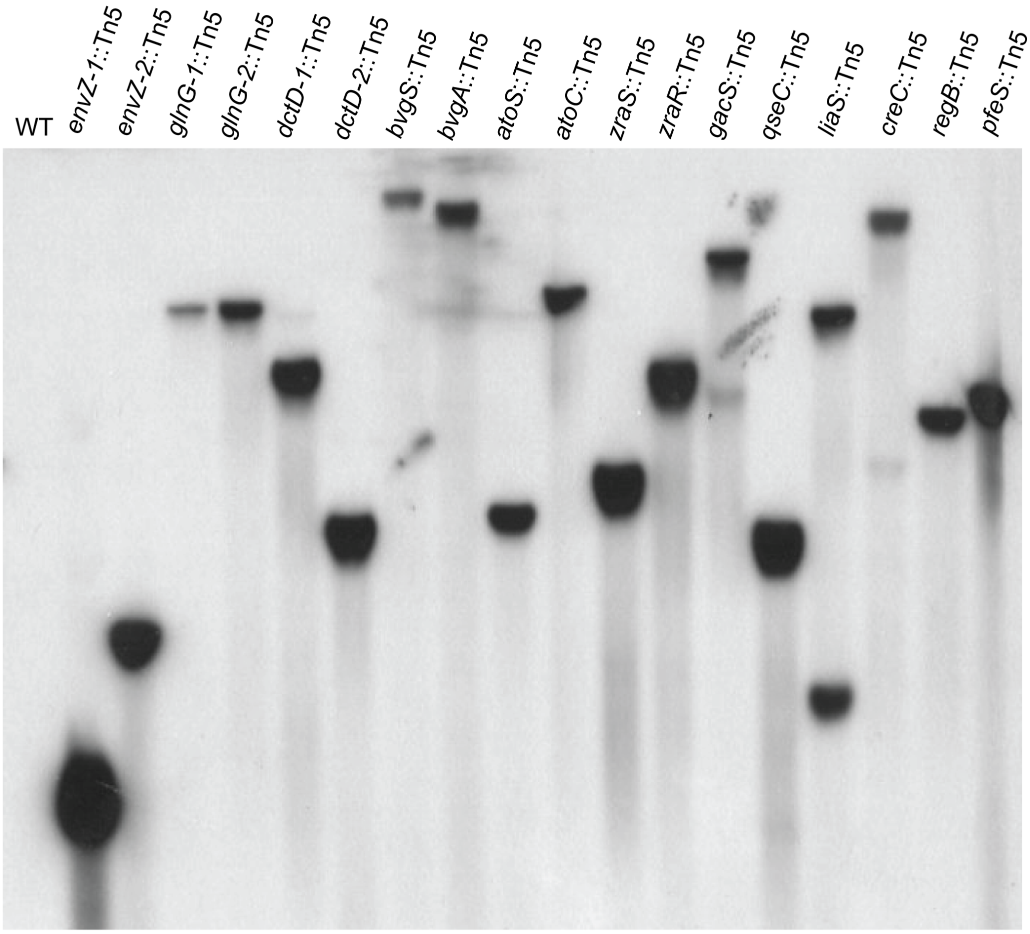

FIGURE S6. Southern blot analysis of EagI-digested genomic DNA of WT and Tn5-inserted mutants of two component systems (TCSs). Wild-type *P. taiwanensis* was used as a negative control. Tn5-inserted mutants of TCS were determined as having a single-copy Tn5 insertion except *lias*::Tn5 mutant. NCBI CDS accession number：*envZ*-1 (GQ77_09865), *envZ*-2 (GQ77_04790), *glnG*-1 (GQ77_01700), *glnG-2* (GQ77_22120), *dctD*-1 (GQ77_18375), *dctD*-2 (GQ77_18060), *bvgS* (GQ77_15860), *bvgA* (GQ77_17140), *atoS* (GQ77_06980), *atoC* (GQ77_12185), *zraS* (GQ77_12195), *zraR* (GQ77_19840), *gacS* (GQ77_17525), *qseC* (GQ77_10465), *liaS* (GQ77_21060), *creC* (GQ77_21495), *regB* (GQ77_04400), *pfeS* (GQ77_17515).

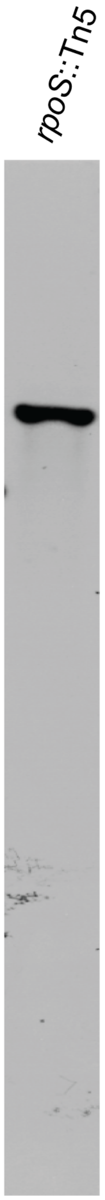

FIGURE S7. Southern blot analysis of EagI-digested genomic DNA of WT and Tn5-inserted mutants of sigma factor *rpoS.* Tn5-inserted mutant of *rpoS* was identified as having a single-copy Tn5 insertion. NCBI CDS accession number: *rpoS* (GQ77_05495).

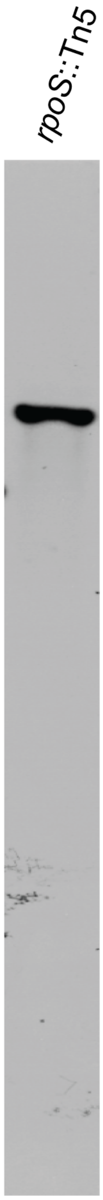

FIGURE S7. Southern blot analysis of EagI-digested genomic DNA of WT and Tn5-inserted mutants of sigma factor *rpoS.* Tn5-inserted mutant of *rpoS* was identified as having a single-copy Tn5 insertion. NCBI CDS accession number: *rpoS* (GQ77_05495).
