# Supplementary material for: Whole Genome Sequencing and Tn5-Insertion Mutagenesis of Pseudomonas taiwanensis CMS to Probe Its Antagonistic Activity Against Rice Bacterial Blight Disease
Source: Int J Mol Sci. 2020 Nov 16;21(22):8639. doi: 10.3390/ijms21228639 (PMC7696974; doi:10.3390/ijms21228639)
Supplement: Supplementary file 1 [file ijms-21-08639-s001.zip › New Table S1.pdf]

TABLE S1. General features of genomes of *P. taiwanensis* and its close-related species *P. entomophila* L48 and *P. putida* KT2400

General features	*P. taiwanensis*[a]	*P. entomophila* L48[b]	*P. putida* KT2440[c]
Function	Biocontrol	Biocontrol	Catabolism of aromatic compounds
Size (Mb)	5.09	5.89	6.18
GC (%)	62.6	64.2	61.5
Genes	4626	5,138	5,786
CDS	4477	5,003	5,564
rRNA	16	22	22
tRNA	68	78	75
Pseudogene	64	34	57
Prophage			
- (incomplete)	0	2	1
- (intact)	3	1	3

GenBank accession numbers: [a]*Pseudomonas taiwanensis*: NCBI accession no. CP011858; [b]*Pseudomonas entomophila* L48: NC_008027.1; [c]*Pseudomonas putida* KT2440: NC_002947.4
