# Supplementary material for: Whole Genome Sequencing and Tn5-Insertion Mutagenesis of Pseudomonas taiwanensis CMS to Probe Its Antagonistic Activity Against Rice Bacterial Blight Disease
Source: Int J Mol Sci. 2020 Nov 16;21(22):8639. doi: 10.3390/ijms21228639 (PMC7696974; doi:10.3390/ijms21228639)
Supplement: Supplementary file 1 [file ijms-21-08639-s001.zip › New Table S2.pdf]

TABLE S2. Specific gene families of *P. taiwanensis* compared to *P. putida* KT2400 and *P. entomophila* L48.

Family[a]	NCBI database no.	Gene product
GP00072	GQ77_17010	Hypothetical protein
	GQ77_17015	Hypothetical protein
	GQ77_17020	Hypothetical protein
	GQ77_17030	Hypothetical protein
	GQ77_17040	Hypothetical protein
GP03502	GQ77_05875	FAD-dependent oxidoreductase
	GQ77_06055	Oxidoreductase
GP03503	GQ77_05990	GntR family transcriptional regulator
	GQ77_06005	GntR family transcriptional regulator
GP03504	GQ77_09115	LuxR family transcriptional regulator
	GQ77_09335	LuxR family transcriptional regulator
GP03505	GQ77_09255	Acyl-CoA dehydrogenase
	GQ77_09280	Acyl-CoA dehydrogenase
GP03506	GQ77_09260	Acyl-CoA dehydrogenase
	GQ77_09275	Acyl-CoA dehydrogenase
GP03507	GQ77_09890	Amidohydrolase
	GQ77_13010	Hypothetical protein
GP03508	GQ77_11120	Histidine kinase
	GQ77_11130	Chemotaxis protein CheY
GP03509	GQ77_15505	Integrase
	GQ77_21185	Integrase
GP03533	GQ77_07380	hypothetical protein
	GQ77_13330	Cupin
GP03534	GQ77_09095	Hypothetical protein
	GQ77_09100	Hypothetical protein
GP03535	GQ77_09885	MFS transporter
	GQ77_12330	MFS transporter
GP03536	GQ77_11140	Fimbrial protein
	GQ77_14145	Hypothetical protein
GP03537	GQ77_19050	Hypothetical protein
	GQ77_19070	Hypothetical protein

[a]The 14 *P. taiwanensis*-specific gene families were identified by comparing the genomic sequences of *P. taiwanensis* and its phylogenetic-related bacteria *P. putida* KT2400 and *P. entomophila* L48 using OrthoMCL.
